# Sleep Disturbances and Autonomic Dysfunction in Patients with Postural Orthostatic Tachycardia Syndrome

**DOI:** 10.3389/fneur.2014.00118

**Published:** 2014-07-07

**Authors:** Julia Mallien, Stefan Isenmann, Anne Mrazek, Carl-Albrecht Haensch

**Affiliations:** ^1^Sleep Unit, Department of Neurology and Neurophysiology, HELIOS Klinikum Wuppertal, University of Witten/Herdecke, Wuppertal, Germany; ^2^Department of Neurology, Kliniken Maria Hilf GmbH, University of Witten/Herdecke, Mönchengladbach, Germany

**Keywords:** PoTS, sleep disturbances, heart rate variability, insomnia, autonomic dysfunction

## Abstract

Many patients with postural tachycardia syndrome (PoTS) suffer from fatigue, daytime sleepiness, and sleeping disturbances. The objective of this study was to compare subjective and objective sleep quality of PoTS patients with a group of healthy controls. All patients completed a Pittsburgh Sleep Quality Index questionnaire and the Epworth Sleepiness Scale. The patients sleep architecture, heart rate, and heart rate variability (HRV) measurements were taken during one night at the sleep laboratorium. All data was collected at the Sleep Unit, at Helios Klinikum Wuppertal. Thirty-eight patients diagnosed with PoTS were compared to 31 healthy controls, matched in age and gender. Patients with PoTS reached significantly higher scores in sleep questionnaires, which means that they were more sleepy and had a lower sleep quality. Polysomnography showed a significantly higher proportion of stage 2 sleep. The results of HRV analysis in different sleep stages confirmed changes in autonomic activity in both groups. PoTS patients, however, showed a diminished variability of the low-frequency (LF) band, high-frequency (HF) band, and LF/HF ratio in different sleep stages. It can therefore be gathered that PoTS could be considered as potential differential diagnosis for sleep disturbances since PoTS patients had a subjective diminished sleep quality, reached higher levels of daytime sleepiness, and showed a higher proportion of stage 2 sleep. PoTS patients showed furthermore a reduction of LF/HF ratio variability in different sleep stages.

## Introduction

The postural tachycardia syndrome (PoTS) is characterized by symptoms of orthostatic intolerance and an excessive increase in heart rate (by ≥30 or to ≥120 bpm) when upright (1–3). Typical complaints of the otherwise healthy patients are palpitations, weakness, blurred vision, chest pain, sweating, dizziness, presyncope, shortness of breath, tremulousness, fatigue, poor nocturnal sleep, and sleep dysfunction including waking up at night repeatedly and night sweating (4). PoTS is a heterogeneous and multifactorial disorder (5). PoTS manifests itself with symptoms of cerebral hypoperfusion and excessive sympathoexcitation but the exact pathophysiology remains as of yet unknown. It has however been found that the pathophysiology of PoTS includes impaired sympathetically mediated vasoconstriction, excessive sympathetic drive, volume dysregulation, and deconditioning, which can all be variously combined.

As mentioned before, some of the chief complaints of PoTS patients are increased daytime sleepiness, fatigue, and diminished quality of night time sleep. In 1921, Swan et al. already mentioned sleep disturbances in relation to PoTS-like symptoms (6). Also Craig et al. and Wood et al. mentioned the incidence of sleep disturbances in their publications regarding neurocirculatory asthenia, which is consistent with today’s definition of PoTS (7, 8). Recent studies confirmed this coherency. In 2007, Thieben et al. investigated 152 medical records which showed that 31.6% of PoTS patients had sleep disturbances and even 48% had severe fatigue (4). Bagai et al. discovered significantly more severe sleep disturbances, higher fatigue levels, and excessive daytime sleepiness in a group of 44 PoTS patients compared to normal controls (*n* = 46) using questionnaires (9). All published evidence is focused on the subjective sleep quality of PoTS patients or actigraphy (10). As of yet, there is no published data regarding objective sleep evaluation of PoTS patients with a polysomnographic (PSG) examination of their sleep quality. The aim of this study is therefore to evaluate subjective and objective sleep quality of PoTS patients compared to a group of healthy controls matched in age and gender. As both sleep and PoTS may be affected by the regulation of the autonomic nervous system (ANS), it can be assumed, that a connection between autonomic dysfunction and sleep disturbance exists. To evaluate this putative coherency, heart rate variability (HRV) was used as an objective measurement for the ANS.

## Materials and Methods

The present clinical trial is a prospective competitive study of patients and controls who presented to the Department of Neurology and Neurophysiology, Helios Klinikum Wuppertal in between June 2010 and September 2011. PoTS patients were mainly examined during the course of their hospitalization. Control subjects were recruited within private contacts and examinations were performed as outpatients. Within the study, we examined 38 patients, which fulfilled conventional criteria of PoTS and 31 healthy control subjects that did not show any symptoms of orthostatic intolerance. All participants completed an informed consent form prior to taking part. Inclusion criteria for the PoTS group were symptoms of orthostatic intolerance plus an increase in heart rate by ≥30 or to ≥120 bpm during upright position. Excluding criteria for both patient groups were all conditions that could affect the autonomous nervous system, such as Parkinson’s disease, diabetes mellitus, alcoholism, hypertension, defects of the thyroid, pheochromocytoma, anemia, dementia, multiple sclerosis, cardiac arrhythmias, or polyneuropathy. Furthermore, patients with psychiatric disorders were excluded. A constant medication or current pregnancy was additional exclusion criterion.

### Autonomic testing

All participants underwent a 70°-tilt table study at the Autonomic Laboratory at Helios Klinikum Wuppertal in order to assess their orthostatic cardiovascular function. All subjects were examined between 7.30 a.m. and 12.30 a.m. Time spent upright on the tilt table varied between 10 and 45 min, depending on the patients tolerance. During this procedure, blood pressure, heart rate, and breathing rate were continuously monitored by a FAN™ computer (Schwarzer, Heilbronn, Germany). Blood pressure was additionally continuously measured with a Portapress device (TNO-TPD Biomedical Instrumentation, Amsterdam, Netherlands) following the method described by Penáz (11, 12). Each participant completed a Valsalva maneuver during which blood pressure and heart rate were monitored. The maneuver was accomplished in supine position (13). After a rest period of 1 min, each participant was asked to expirate against a pressure of 20–40 mmHg for approximately 15 s. Valsalva ratio and changes of blood pressure were measured by the FAN™ device (Schwarzer GmbH, Munich, Germany). The FAN™ device records HRV based on the standard recording techniques and algorithms used in autonomic function studies (14). The FAN™ has an interface box for the recording of ECG, breathing frequency, and expiratory pressure, e.g., during Valsalva maneuver. ECG and breathing frequency were digitized with a sampling rate of 500 Hz; where the expiratory pressure could be recorded with the limits of 0–100 mm mercury. The interface also offers the opportunity to record one analog external signal of blood pressure from an external monitor (digitizing rate 500 Hz).

### Polysomnography

All participants were monitored in the Sleep Unit, Helios Klinikum Wuppertal that fulfills the requirements by the German Society of Sleep Science and Sleep Medicine (DGSM). As a standard procedure, all patients stayed overnight and were attended with PSG. The recordings were evaluated using “Alice 5” – PSG soft – and hardware (Heinen & Loewenstein, Bad Ems, Germany). The PSG recording included six channel electroencephalogram (EEG) (including frontal, central, occipital bilateral referencing to right and left mastoid), right and left electro-oculography, bilateral surface EMG of mentalis and of right and left tibialis anterior muscles, 2 channel ECG, oronasal thermistor, thoracic and abdominal respirograms, absolute position transducer larynx microphone, and oxygen saturation. The subjects reported to the sleep unit at 8.00 p.m. Monitoring was performed for at least 6 h (10 p.m. until 5 a.m.) All patients were monitored over the period of one night.

### Data analysis

Sleep data were staged visually according to the rules of Rechtschaffen & Kales and furthermore according to the criteria of the American Association of Sleep Medicine using 30 s time frames, by two independent investigators (15, 16). PSG parameters like mean heart rate in NREM and REM sleep, Total sleep time (TST), total in bed time (TIBT), sleep efficiency (TST/TIBT), sleep onset latency (minutes), REM latency (minutes), quantity of stage 2 (%), slow wave sleep (%), and REM (%) sleep were analyzed. Arousals were defined as abrupt changes in EEG pattern with return to alpha or theta frequency, lasting between 3 and 10 s and were also analyzed according to the criteria of the American Association of Sleep Medicine (16).

### Heart rate variability

Heart rate variability data were detected using ECG signals: 5 min segments of artifact-free ECG data during sleep stage 2, slow wave sleep, and REM sleep were selected for analyses (17). ECG lines were extracted from Alice 5 software and transferred to the *fan* computer by an ASCI file. All ECG lines were visually controlled for artifacts and extra-systoles. HRV was automatically analyzed with ASCI 2 at a sampling rate of 500 Hz by the Fan device software according to the “Recommendations of the International Federation of Clinical Physiology for the Practice of Clinical Neurophysiology” (18, 19). In short, the ECG signal was digitized at a sample rate of 500/s. Respiration was monitored by registration of chest movements. A fast Fourier transform (FFT)-based algorithm was used for the spectral analysis of HRV. The RR intervals were converted to 4 Hz and an exact Hamming window was applied for FFT. The power in frequency ranged from low frequencies (LF: 0.04–0.15 Hz) to high frequencies (HF: 0.15–0.40 Hz). Spectral analysis allowed a differentiation of sympathetic and parasympathetic activation, which were related to a LF and a HF component of the HRV signal, respectively. LF band reflected a combination of sympathetic and parasympathetic influences and baroreflex function. HF band reflected parasympathetic activity. The resulting LF/HF ratio as a quantitative index of the sympathovagal balance was calculated.

### Epworth Sleepiness Scale

The Epworth Sleepiness Scale (ESS) is a standardized questionnaire, which documents subjective daytime sleepiness. It is focused on eight specific daily situations including sitting, reading, watching television, passive sitting (e.g., in a theater), co-driving, while stopping and driving (e.g., on a traffic light), afternoon rest, sitting while having a conversation and after lunch (without the consumption of alcohol). Patients were asked to rank sleepiness in these situations on a scale of 0–3 points. Total possible maximum would have been 24 points. Prior studies showed that an ESS Score over 10 is related to enhanced daytime sleepiness (20).

### Pittsburgh Sleep Quality Index

The Pittsburgh Sleep Quality Index (PSQI) is a standardized questionnaire to retrospectively document subjective sleep quality during the past for weeks (21). Its seven categories consist of subjective sleep quality, sleeping time, sleep latency, intake of sleep medication, sleep efficiency, sleep disturbances, and daytime sleepiness. Each category can score up to three points. Total maximum score would be 21 points. Participants could be classified in three groups. A score of ≤5 indicates good quality of sleep, a score ≥5 and ≤10 indicated minor quality of sleep, and a score of ≥10 correlated with chronic sleeping disorders.

### Ethics of investigations

All clinical studies conformed with the principals of the declaration of Helsinki. A positive voting of the Ethics committee of the University of Witten/Herdecke had been obtained prior to data collection.

### Statistical methods

All statistical analyses were performed using SPSS for Windows v. 20.0 (SPSS Inc., Chicago, IL, USA) with assistance of the statistician Dr. Lange. Descriptive statistics are presented as mean ± SD or median [plus interquartile range (>IQR)]. To compare independent variables Mann–Whitney-*U* or Fisher Tests were performed. For more than two dependant variables, Friedman test was used. All tests were two-tailed. Statistical significance was set at *p* = 0.05.

## Results

### Demographics

Sixty-nine persons in total were examined, 38 patients with PoTS, and 31 healthy controls. PoTS patients were predominantly female (74%). They were between 18 and 41 years of age, with a mean age of 25.3 ± 7 years (females: 24.4 ± 6.2 years, males: 27.8 ± 8.1 years). Age and gender of control subjects was adapted to the PoTS group not resulting in significant differences (age *p* = 0.183, gender *p* = 0.518). Thirty-one healthy participants were assigned to the control group, 20 females (65%) and 11 males. The mean age of this group was 26.2 ± 6.3 years (females: 25.7 ± 5.8 years, males: 27.2 ± 7.5 years). Controls were recruited within a university setting.

### Autonomic testing

Head-up tilt table testing showed an increase in heart rate of PoTS patients with a median of 38.99 bpm (30.00–43.00). This was significantly higher than in controls (*p* = 0.000), who showed a median in heart rate increase by 15.00 bpm (12.00–25.00). No significant changes in blood pressure were found in both groups. During Valsalva maneuver, blood pressure was continuously measured. PoTS patients had an increase in systolic blood pressure during phase 4 of the maneuver with a median of 27.00 (16.00–44.00) mmHg. Controls, however, showed an increase systolic pressure with a median of 21.00 (15.00–26.00) mmHg, resulting in a statistically significant difference in between groups (*p* = 0.019). PoTS patients showed furthermore significantly higher parameters in the Valsalva ratio (1.92 vs. 1.75; *p* = 0.0036).

### Subjective sleep quality and sleepiness

Twenty-five PoTS patients and 31 control subjects answered PSQI and ESS questionnaires. The maximum PSQI score reached by PoTS patients was 17, controls reached a maximum score of 12. Ten PoTS patients accomplished a score ≥10 (40%). Whereas only one participant of the control group scored ≥10. Compared to controls, PoTS patients reached a significantly higher score in PSQI [PoTS in median: 8 (4–13); where controls had a median of 4 (3–5) *p* = 0.01]. PoTS patients reach higher scores in ESS than controls, but the median did not exceed the cut-off of 10 [PoTS in median: 6 (3–9); where Controls had a median of 3 (1–5) *p* = 0.01]. The maximum score in the PoTS group was 17, participants in the control group reached a maximum score of 7. 24% of PoTS patients reached a score over 10, while none of the control subjects did, however, this did not reach statistic significance (*p* = 0.088).

### Polysomnography

The mean heart rate during different sleep stages is shown in Table 1. PoTS patients have a higher mean heart rate in both sleep stages, NREM and REM, but there is no significant difference between the two groups (Table 1). Parameters that describe the sleep efficiency did not differ significantly in both groups (Table 2). Both groups slept well, with a total sleeping time in median of 359.00 (324.50–381.50) minutes (PoTS) and 350.00 (332.50–375.50) minutes (controls) and sleep efficiencies in median of 96.03 (91.54–98.46)% (PoTS) and 95.56 (93.06–98.38)% (controls). PoTS patients have a significantly higher percentage of stage 2 sleep than controls (42.45 vs. 36.70%; *p* = 0.017). They also show a slightly lower proportion of slow wave sleep (39.25 vs. 41.30%). PoTS patients show a higher proportion of REM sleep (15.10 vs. 12.90%). Furthermore, four of the PoTS patients did not even reach REM sleep stage during the whole night.

**Table 1:** **Heart rate during NREM and REM sleep presented in median with its interquartile range**.

Variable	PoTS	Controls	*p* Value
Mean heart rate NREM	61.60 (56.10 – 68.00)	59.60 (54.90 – 68.90)	0.859
Mean heart rate REM	66.75 (57.50 – 70.20)	63.20 (58.50 – 70.30)	0.864

**Table 2:** **Parameters of sleep efficiency and sleep architecture presented in median with its interquartile range**.

Variable	PoTS	Controls	*p* Value
Total in bed time (TIBT) (minutes)	401.50 (385.00 – 423.00)	402.00 (386.00 – 423.00)	0.777
Sleep period time (SPT) (minutes)	372.00 (349.50 – 398.50)	368.50 (353.50 – 396.00)	0.847
Total sleep time (TST) (minutes)	359.00 (324.50 – 381.50)	350.00 (332.50 – 375.50)	0.828
Sleep efficiency (TST/SPT) (%)	96.03 (91.54 – 98.46)	95.56 (93.06 – 98.38)	0.981
TST/TIBT (%)	90.52 (82.95 – 93.78)	87.26 (84.54 – 91.72)	0.546
Sleep latency (minutes)	21.25 (16.50 – 29.00)	24.50 (17.00 – 38.00)	0.393
REM latency (minutes)	138.50 (64.00 – 192.50)	122.50 (84.50 – 227.50)	0.173
Sleep stage 2 (%)	42.45 (35.30 – 48.60)	36.70 (30.60 – 43.00)	0.017
Slow wave sleep (%)	39.25 (31.40 – 47.90)	41.30 (33.00 – 50.90)	0.337
REM (%)	15.10 (6.90 – 19.80)	12.90 (9.90 – 18.70)	0.537

### Arousals

Arousals during night time sleep was measured and manually assessed. It was defined as an increase in EEG frequency lasting at least over a period of 3–10 s. In PoTS patients, there was a total of 41.00 (33.00–50.00) arousals measured, while control subjects showed only 37.00 (27.00–66.00; n.s.). Three of the PoTS patients showed 15 or more periodic leg movements (PLM) per hour, which is consistent with nocturnal movement disorder. One of the PoTS patients showed an index as severe as 54/h. None of the control subjects exhibited more than 11 PLM/h. Furthermore, one of the PoTS patients was diagnosed with obstructive sleep apnea with a REM-AHI of 41.5/h and AHI 10.5/h, showing a mean of nine respiratory arousals per hour. In control subjects, no respiratory arousals could be identified.

### Heart rate variability

#### Low-frequency band

No significant difference in the LF band component between the two groups was found (Table 3). There is no significant increase or decrease of the LF band during different sleep stages in PoTS patients, whereas individuals in the control group had a significant decrease in sleep stage 2 to slow wave sleep (*p* = 0.014). In a summary, these results showed a reduction of variability of the LF band during different sleep stages in PoTS patients.

**Table 3:** **LF band power (ms^2^/s.) across different sleep stages in median with its interquartile range**.

Variables	PoTS	Controls	*p* Value
Sleep stage 2	336.68 (149.52 – 641.58)	498.09 (185.81 – 689.04)	0.392
Slow wave sleep	224.69 (118.87 – 589.83)	227.18 (89.03 – 663.87)	0.968
REM	365.20 (155.00 – 591.14)	285.77 (165.65 – 562.34)	0.767

#### High-frequency band

There was no statistically significant difference between HF bands in between PoTS patients and controls (Table 4). In controls, there were significant differences of HF band between stage 2 and slow wave sleep (*p* = 0.018) and stage 2 and REM (*p* = 0.006), but there was no statistical difference between HF band of slow wave sleep and REM. In PoTS patients, there was no statistical difference of HF band increase or decrease between sleep stages 2, slow wave sleep, and REM. HF band of PoTS patients did not vary in between sleep stages as much as control subjects.

**Table 4:** **HF band power (ms^2^/s) across different sleep stages in median with its interquartile range**.

Variables	PoTS	Controls	*p* Value
Sleep stage 2	333.10 (188.72 – 600.79)	250.91 (155.79 – 969.79)	0.975
Slow wave sleep	342.34 (181.68 – 621.52)	237.18 (166.18 – 678.45)	0.386
REM	227.39 (112.47 – 749.35)	176.39 (62.62 – 389.17)	0.192

### LF/HF ratio

There was no statistically significant difference between the two study groups (Table 5). PoTS patients showed a decrease of LF/HF ratio from stage 2 to stage 4 (n.s.) and an increase to stage REM (n.s.). In controls, there were significant changes in HRV in between sleep stages. LF/HF ratio was higher in sleep stage 2 than in slow wave sleep (*p* = 0.265), and in both stage 2 (*p* = 0.002) and slow wave sleep (*p* = 0.002) were significantly lower compared to REM sleep. PoTS patients show a reduction of LF/HF ratio variability in different sleep stages.

**Table 5:** **LF/HF ratio across different sleep stages in median with its interquartile range**.

Variables	PoTS	Controls	*p* Value
Sleep stage 2	0.82 (0.47 – 1.69)	0.93 (0.58 – 1.99)	0.542
Slow wave sleep	0.64 (0.36 – 1.41)	0.81 (0.46 – 2.21)	0.386
REM	1.14 (0.51 – 2.63)	1.52 (0.87 – 3.91)	0.187

## Discussion

In order to assess subjective and objective sleep quality of PoTS patients, this study considered a group of 38 PoTS patients and compared them to a group of 31 healthy controls matched in age and gender. All patients included met standard diagnostic criteria for PoTS. Basically, the results obtained in this study confirmed current literature, i.e., PoTS patients showed physiological autonomic control of the baroreflex arc as shown by the Valsalva maneuver with diminished subjective sleep quality and increased daytime sleepiness. For the first time, it was demonstrated that in PoTS patient the macrostructure of sleep is physiological by means of PSG recording. Autonomic testing showed a significantly higher Valsalva ratio in PoTS patients as compared to normal subjects. This conforms to former studies of Sandroni et al. who found a higher Valsalva ratio in PoTS patients and ascribed this to an increased β-adrenergic tone (22). Furthermore, there was a higher increase in systolic blood pressure in PoTS patients during Valsalva maneuver compared to control subjects, which also reflected the findings of Sandroni et al. (22). Forty percent of PoTS patients in this study suffered from subjective diminished sleep quality as evaluated by a standardized PSQI questionnaire. Furthermore, 24% reached an ESS score above 10, which reflected excessive daytime sleepiness in those patients. Our study confirmed the results of Thieben et al., who reported that 31.6% of a cohort of 152 PoTS patients suffered from sleep disturbances and even 48.0% that complained about fatigue (4). Bagai et al. reported 51% PoTS patients (*n* = 44) with excessive daytime sleepiness (9). One could expect a coherency of diminished subjective sleep quality, increased daytime sleepiness, and diminished sleep efficiency. In our study, PSG parameters showed no differences in sleep efficiency or frequency of arousals between both groups. One of the PoTS patients was diagnosed with sleep apnea and treated with CPAP (ESS score 2) whilst two PoTS patients showed increased PLM during night, these patients also showed ESS scores over 10, which could be due to PLMs. The significance of these findings needs further evaluation. However, sleep architecture showed a significantly higher proportion of sleep stage 2 in patients with PoTS. This phenomenon could also be detected in patients with insomnia and points out the evidence of objective primary sleep disorders in PoTS patients. Slow wave sleep percentage was slightly higher in control subjects (39.25 vs. 41.30%; n.s.), but not statistically significant. There was no significant difference in the proportion of REM sleep between the two groups. However, four of the PoTS patients did not even reach REM sleep stage throughout the night.

Spectral analyses of HRV data during different sleep stages (sleep stage 2, slow wave sleep, and REM) demonstrated characteristic sleep stage-related changes in ANS activity in control subjects (23–27). Parasympathetic activity increased with synchronization of sleep while LF/HF ratio which presents sympathovagal balance decreased. In REM sleep, LF/HF ratio increased, which indicates a sympathetic dominance. Separate analyses of LF band, HF band, and LF/HF ratio showed stage-related differences between the two patient groups. In control subjects, LF band showed a significant decrease from sleep stage 2 to slow wave sleep, which could not be detected in patients with PoTS. Furthermore in control subjects, HF band showed a significant decrease from sleep stage 2 to slow wave sleep and also from slow wave sleep to REM sleep. In PoTS patients, HF band did not vary to the same extent. Median values of the LF/HF ratio appeared to be consistently lower in PoTS patients. With reference to LF/HF ratio, there was a significant increase from sleep stage 2 to REM sleep and also from slow wave sleep to REM sleep in control subjects. In PoTS patients, no significant stage-related change in LF/HF ratio was seen. This phenomenon leads to the conclusion that PoTS patients have an altered HRV during night. In some PoTS patients, cardiac dysautonomic neuropathy with reduced myocardial 123I-meta-iodobenzylguanidine uptake was reported, which could also be a reason for diminished HRV in different sleep stages (28, 29). An altered state dependent modulation of HRV may not only derive from cardiac dysautonomic neuropathy, but also from a hyperadrenergic state (5).

As a limitation of the present study, the results of sleep efficiency, sleep architecture, and arousal frequency should be interpreted carefully since they could be affected by a “first night effect” (30) due to the lack of an adaption night. However, according to Israel et al., HRV is not affected by the “first night effect” (31). With regard to the measurement of arousals, the present study is limited by the consideration of changes in EEG patterns only. If there are other sorts of arousals, which would only affect cardiorespiratory parameters, like autonomic arousals presumably do, they would have not been evaluated by the present trial. In fact, there is no proper definition to classify autonomic arousals to this date. Future studies into this field could evaluate autonomic arousal, since it may affect sleep architecture and cause sleep disturbances in PoTS patients. Very recently Sasai et al. found that an elevation of sympathetic nervous system activity and mean frequency fluctuation in an HF band can occur several seconds before the period of PLMs. Therefore, specific analysis of HRV in PLM period could be an interesting aspect for future research. Our results are probably unaffected by autonomic fluctuation prior to PLMs, since analyzed HRV periods were copied to the ECG at least 10 min before or after an arousal occurred. The presence of PLMS or OSA(S) (AHI 10.5/h) may be a confounding factor for analysis. Another theory, which is not evaluated in the present study, is the coherency of sleep difficulties and anxiety in PoTS patients. It is conceivable that this could be an alternative cause of sleeping disorders, as a connection between anxiety disorders and sleep disturbances in other patients as already reported by Soehner et al. (32). On the other hand, in a previous study, it has been found that an increase of propensity to anxiety disorders existed in PoTS only when questionnaires were used that included autonomic items (33). For further research, PoTS patients could be divided into two groups via PSQI and ESS scores, thus autonomic parameters in PoTS patients could be compared with and without subjective sleeping disorder/sleepiness. From a statistical point of view, a large number of variables were tested without controlling for type I error (e.g., Bonferroni approach). The study is also restricted by performing it only in the Helios Klinikum Wuppertal and results may not be transmitted to other settings due to, e.g., other ethnicities in other clinical setting.

## Conclusion

Postural tachycardia syndrome patients had a subjective diminished sleep quality, reached higher levels of daytime sleepiness, and showed a diminished proportion of sleep stage 2. Since all these symptoms can be also seen in patients with insomnia, PoTS should be included as a potential differential diagnosis of insomniacs. PoTS patients showed normal sleep when objectively evaluated by PSG. Furthermore, analyses of HRV during different sleep stages showed altered HRV during different sleep stages in PoTS. Sleep physicians should be aware of sleep disturbances associated with this form of orthostatic intolerance.

## Conflict of Interest Statement

The authors declare that the research was conducted in the absence of any commercial or financial relationships that could be construed as a potential conflict of interest.

## References

[1] Brady PA Low PA Shen WK Inappropriate sinus tachycardia, postural orthostatic tachycardia syndrome, and overlapping syndromes. Pacing Clin Electrophysiol (2005) 28(10):1112–21 10.1111/j.1540-8159.2005.00227.x 16221272

[2] Grubb BP Kanjwal Y Kosinski DJ The postural tachycardia syndrome: a concise guide to diagnosis and management. J Cardiovasc Electrophysiol (2006) 17(1):108–12 16426415 10.1111/j.1540-8167.2005.00318.x

[3] Mathias CJ Low DA Iodice V Owens AP Kirbis M Grahame R Postural tachycardia syndrome – current experience and concepts. Nat Rev Neurol (2012) 8(1):22–34 10.1038/nrneurol.2011.187 22143364

[4] Thieben MJ Sandroni P Sletten DM Benrud-Larson LM Fealey RD Vernino S Postural orthostatic tachycardia syndrome: the Mayo clinic experience. Mayo Clinic Proc (2007) 82(3):308–13 10.4065/82.3.308 17352367

[5] Benarroch EE Postural tachycardia syndrome: a heterogeneous and multifactorial disorder. Mayo Clinic Proc (2012) 87(12):1214–25 10.1016/j.mayocp.2012.08.013 PMC3547546 23122672

[6] Swan JM An analysis of ninety cases of functional disease in soldiers. Trans Am Climatol Clin Assoc (1921) 37:44–72 21408783 PMC2307433

[7] Wood P Da Costa’s syndrome (or effort syndrome). Lecture II. Br Med J (1941) 1(4195):805–11 20783683 10.1136/bmj.1.4195.805 PMC2161993

[8] Craig HR White PD Etiology and symptoms of neurocirculatory asthenia. Arch Intern Med (1934) 53:645–8 10.1001/archinte.1934.00160110002001

[9] Bagai K Song Y Ling JF Malow B Black BK Biaggioni I Sleep disturbances and diminished quality of life in postural tachycardia syndrome. J Clin Sleep Med (2011) 7(2):204–10 21509337 PMC3077350

[10] Bagai K Wakwe CI Malow B Black BK Biaggioni I Paranjape SY Estimation of sleep disturbances using wrist actigraphy in patients with postural tachycardia syndrome. Auton Neurosci (2013) 177(2):260–5 10.1016/j.autneu.2013.02.021 23538032 PMC3700681

[11] Peñáz J Photoelectric measurement of blood pressure, volume and flow in the finger. Digest of the 10th International Conference on Medical and Biological Engineering Dresden: International Federation for Medical and Biological Engineering (1973). 104 p.

[12] Wesseling KH de Wit B van der Hoeven GMA van Goudoever J Settels J Physical, calibrating finger vascular physiology for Finapres. Homeostasis (1995) 36(2–3):67–82

[13] Singer W OpferGgehrking TL McPhee BR Hilz MJ Low PA Influence of posture on the Valsalva manoeuvre. Clin Sci (2001) 100(4):433–40 10.1042/CS20000208 11256984

[14] Haegele-Link S Claus D Ducker S Vogt T Birklein F Evaluation of the autonomic nervous system using the FAN device – range of normal and examples of abnormal. Open Neurol J (2008) 2:12–9 10.2174/1874205X00802010012 19018302 PMC2577935

[15] Rechtschaffen A Kales A A Manual of Standardized Terminology, Techniques and Scoring System for Sleep Stages of Human Subjects. Los Angeles: Brain Information Service, Brain Research Institute, UCLA (1968).

[16] Iber C Ancoli-Israel S Chesson A Quan SF The AASM Manual for the Scoring of Sleep and Associated Events: Rules, Terminology and Technical Specifications. 1st ed Westchester, IL: American Academy of Sleep Medicine (2007).

[17] Heart rate variability: standards of measurement, physiological interpretation, and clinical use. Task Force of the European Society of Cardiology and the North American Society of Pacing and Electrophysiology. Eur Heart J (1996) 17(3):354–81 10.1093/oxfordjournals.eurheartj.a014868 8737210

[18] Novak V Saul JP Eckberg DL Task Force report on heart rate variability. Circulation (1997) 96(3):1056–7 9264529

[19] Heart rate variability: standards of measurement, physiological interpretation and clinical use. Task Force of the European Society of Cardiology and the North American Society of Pacing and Electrophysiology. Circulation (1996) 93(5):1043–65 10.1161/01.CIR.93.5.1043 8598068

[20] Johns MW A new method for measuring daytime sleepiness: the Epworth sleepiness scale. Sleep (1991) 14(6):540–5 1798888 10.1093/sleep/14.6.540

[21] Buysse DJ Reynolds CF III Monk TH Berman SR Kupfer DJ The Pittsburgh Sleep Quality Index: a new instrument for psychiatric practice and research. Psychiatry Res (1989) 28(2):193–213 10.1016/0165-1781(89)90047-4 2748771

[22] Sandroni P Novak V Opfer-Gehrking TL Huck CA Low PA Mechanisms of blood pressure alterations in response to the Valsalva maneuver in postural tachycardia syndrome. Clin Auton Res (2000) 10(1):1–5 10.1007/BF02291382 10750636

[23] Vanoli E Adamson PB Ba L Pinna GD Lazzara R Orr WC Heart rate variability during specific sleep stages. A comparison of healthy subjects with patients after myocardial infarction. Circulation (1995) 91(7):1918–22 10.1161/01.CIR.91.7.1918 7895347

[24] Scholz UJ Bianchi AM Cerutti S Kubicki S Vegetative background of sleep: spectral analysis of the heart rate variability. Physiol Behav (1997) 62(5):1037–43 10.1016/S0031-9384(97)00234-5 9333197

[25] Baharav A Kotagal S Gibbons V Rubin BK Pratt G Karin J Fluctuations in autonomic nervous activity during sleep displayed by power spectrum analysis of heart rate variability. Neurology (1995) 45(6):1183–7 10.1212/WNL.45.6.1183 7783886

[26] Vaughn BV Quint SR Messenheimer JA Robertson KR Heart period variability in sleep. Electroencephalogr Clin Neurophysiol (1995) 94(3):155–62 10.1016/0013-4694(94)00270-U 7536150

[27] Toscani L Gangemi PF Parigi A Silipo R Ragghianti P Sirabella E Human heart rate variability and sleep stages. Ital J Neurol Sci (1996) 17(6):437–9 10.1007/BF01997720 8978452

[28] Haensch CA Lerch H Jigalin A Schlemmer H Isenmann S Cardiac denervation in postural tachycardia syndrome. Clin Auton Res (2008) 18(1):40–2 10.1007/s10286-007-0442-z 17898925

[29] Haensch CA Lerch H Schlemmer H Jigalin A Isenmann S Cardiac neurotransmission imaging with 123I-meta-iodobenzylguanidine in postural tachycardia syndrome. J Neurol Neurosurg Psychiatry (2010) 81(3):339–43 10.1136/jnnp.2008.168484 19687022

[30] Scholle S Scholle HC Kemper A Glaser S Rieger B Kemper G First night effect in children and adolescents undergoing polysomnography for sleep-disordered breathing. Clin Neurophysiol (2003) 114(11):2138–45 10.1016/S1388-2457(03)00209-8 14580612

[31] Israel B Buysse DJ Krafty RT Begley A Miewald J Hall M Short-term stability of sleep and heart rate variability in good sleepers and patients with insomnia: for some measures, one night is enough. Sleep (2012) 35(9):1285–91 10.5665/sleep.2088 22942507 PMC3413806

[32] Soehner AM Harvey AG Prevalence and functional consequences of severe insomnia symptoms in mood and anxiety disorders: results from a nationally representative sample. Sleep (2012) 35(10):1367–75 10.5665/sleep.2116 23024435 PMC3443763

[33] Wagner C Isenmann S Ringendahl H Haensch CA Anxiety in patients with postural tachycardia syndrome (PoTS). Fortschr Neurol Psychiatr (2012) 80(8):458–62 10.1055/s-0031-1299106 22692879

